# Associations between the maternal circulating lipid profile in pregnancy and fetal imprinted gene alleles: a cohort study

**DOI:** 10.1186/s12958-018-0399-x

**Published:** 2018-08-29

**Authors:** Clive J. Petry, Albert Koulman, Liangjian Lu, Benjamin Jenkins, Samuel Furse, Philippa Prentice, Lee Matthews, Ieuan A. Hughes, Carlo L. Acerini, Ken K. Ong, David B. Dunger

**Affiliations:** 1 Department of Paediatrics, University of Cambridge, Box 116, Cambridge Biomedical Campus, Hills Road, Cambridge, CB2 0QQ UK; 2 Medical Research Council Human Nutrition Research, Cambridge, UK; 3 The Institute of Metabolic Science, University of Cambridge, Cambridge, UK; 4 Khoo Teck Puat-National University Children’s Medical Institute, National University Hospital, National University Health System, Singapore, Singapore; 5 Medical Research Council Epidemiology Unit, University of Cambridge, Cambridge, UK

**Keywords:** Lipidomics, Lipid profiling, Triglycerides, Gestational diabetes, Development, Parent-of-origin

## Abstract

**Background:**

Imprinted genes, which are expressed in a parent of origin-specific manner, are thought to mediate the genetic priorities of each parent in pregnancy. Recently we reported that some fetal imprinted gene variants are associated with maternal glucose concentrations and blood pressures in pregnancy. We suggest that the conflict between the effects of paternal and maternal transmitted genes starts at conception and may already be evident in measures of maternal metabolism in early pregnancy, before gestational diabetes is manifest.

**Methods:**

Lipid fractions in maternal non-fasting serum collected around week 15 of pregnancy were profiled using direct infusion mass spectrometry in a subset Discovery Cohort (*n* = 200) of women from the Cambridge Baby Growth Study using direct infusion mass spectrometry. Associations between 151 haplotype-tag fetal polymorphisms in 16 imprinted genes and lipids were determined using partial least squares discriminant analysis. Variable importance in projection scores were used to identify those lipid species that contribute most to the underlying variation in the lipid profile and the concentrations of these species tested for associations with fetal imprinted gene alleles using linear regression. In an internal Validation Cohort (*n* = 567 women from the same cohort) the lipid fraction was profiled using liquid chromatography-mass spectrometry and tested for associations with the same fetal imprinted gene variants as above, followed by meta-analysis of associations from the Discovery and Validation Cohorts.

**Results:**

The most significant associations were between a monounsaturated triglyceride (44:1) and both paternally-transmitted fetal *H19* rs7950932 (*R* = 0.14, *p* = 2.9 × 10^− 3^, *n* = 386) and maternally-transmitted fetal *FAM99A* rs7131362 (*R* = 0.18, *p* = 6.2 × 10^− 3^, *n* = 351; association with maternal-untransmitted allele *R* = 0.08, *p* = 0.07, *n* = 328). This same triglyceride isoform was also associated with subsequent week 28 fasting glucose concentrations (*R* = 0.09, *p* = 9.9 × 10^− 3^, *n* = 673) and homeostasis model assessment of insulin resistance (R = 0.09, *p* = 0.01, *n* = 664).

**Conclusions:**

Fetal imprinted genes may influence maternal circulating clinically relevant triglyceride concentrations early in pregnancy.

## Background

The Kinship (or Conflict) Hypothesis [1, 2] suggests that paternally-expressed fetal imprinted genes will tend to increase fetal growth whereas maternally-expressed genes will tend to restrain it. It has been suggested that this is achieved through modifying fetal and placental nutritional demand and supply [3]. This is supported by evidence that polymorphic variation in the fetal genome, particularly in fetal growth genes, could lead to alterations in maternal metabolism in pregnancy [4]. This long-standing hypothesis suggests that imprinted genes mediate the separate reproductive genetic priorities of the mother and the father in pregnancies [1, 2], through their parent-of-origin-mediated expression. Which parentally-transmitted copy of the gene is expressed, and which is repressed, is governed by the particular gene and the tissue in question, as well as the stage of development. We suggested that in altering both maternal metabolism and physiology, variation in the fetal genome could lead to an alteration in maternal glucose concentrations and blood pressures, leading to a change in the risk of individuals developing adverse conditions of pregnancy such as gestational diabetes (GDM) and gestational hypertension [5, 6]. Following up the equivalent results that we found in an *H19* knockout mouse model [7], in humans we observed that paternally-transmitted fetal alleles in the *IGF2* gene were associated with maternal glucose concentrations in late pregnancy [8] and GDM when contributing towards fetal imprinted gene allele scores [9]. We have also found other fetal imprinted gene variants that are associated with maternal blood pressure in pregnancy [10]. None of the other maternal circulating nutrient groups that could contribute to fetal growth and development appear to have been tested for associations with polymorphic variation in the fetal genome thus far. This includes lipids such as triglycerides.

In addition to the influence of the mother’s dietary intake, concentrations of triglycerides in the maternal circulation during pregnancy appear to be able to be influenced by polymorphic variation in the maternal genome (e.g. [11]). As well as with maternal circulating lipid concentrations, associations with fatty acids in cord blood have been reported for both maternal and fetal polymorphisms (e.g. [12]). Triglycerides in the maternal circulation appear to have a number of positive effects on the pregnancy including being: (a) a mobile energy source, (b) a reservoir of fatty acids in the mother and (c) an important contributor to fetal growth and potentially newborn development (via the de novo biosynthesis of lipids and triglycerides in mammary glands) [13]. However they have also been linked to negative effects of the high abundance of triglycerides in the maternal circulation, e.g. pre-eclampsia, pre-term birth, macrosomia and maternal cardiovascular disease later in life. Triglycerides are strongly associated with fetal growth in women with GDM [14], and changes in their abundance in their circulating concentrations and isoform profile can precede the development of adverse conditions of pregnancy [15]. Whilst acknowledging that there are many factors in pregnancy that may affect the abundance of lipids in the maternal circulation, including variation in the maternal genome, given the potential influence of these triglyceride species on fetal growth and development it seems reasonable to suggest that polymorphic variation in the fetal genome may have its own influence on maternal circulating lipid levels in pregnancy. This could contribute to the regulation of fetal growth and development.

Previously we have found associations between paternally-transmitted fetal *IGF2* and late pregnancy maternal glucose concentrations [8, 9]. This led us to the hypothesis that the interaction between the fetal genotype and maternal metabolism occurs early in pregnancy, when GDM is less well defined. We therefore used mass spectrometry-based lipid profiling to identify metabolic signals and test the hypothesis that fetal variants in imprinted genes are associated with the profile of maternal serum lipids during the first half of pregnancy.

## Methods

### Cohort and sample selection

The prospective and longitudinal Cambridge Baby Growth Study recruited mothers (and their partners and offspring) attending ultrasound clinics during early pregnancy at the Rosie Maternity Hospital, Cambridge, U.K. between 2001 and 2009 [8–10, 16]. Where possible venous blood (non-fasting) was collected from mothers at recruitment (at the pregnancy booking clinic, around 15 weeks of gestation). After allowing time for clotting, these samples were centrifuged at 3000 g for 10 min. and the serum separated and stored at − 80 °C until they were used. In total, 845 DNA triads were collected from the families of 1074 mothers recruited to the study for whom week 28 75 g oral glucose tolerance test (OGTT) data were collected for GDM studies [8, 9]. Blood and/or mouth swab samples for DNA extraction were collected from the father and the offspring *post-partum*. In this cohort 96.9% of the offspring were Caucasian, 0.8% were mixed race, 0.6% were Black (African or Caribbean), 0.8% were Oriental and 0.9% were Indo-Asian.

The current analysis was restricted to 767 women for whom we had both DNA family triad samples available and serum samples from around week 15 of pregnancy. To be of use in a range of studies, 200 of these samples (“the Discovery Cohort”) were specifically chosen to have broadly equal: (1) numbers drawn from tertiles of the venous glucose concentration 60 min. post glucose load at the week 28 OGTT, (2) proportions of pregnancies where the baby exhibited “normal”, “catch-up” or “catch-down” growth (defined as > − 0.67 and < + 0.67, > + 0.67, and < − 0.67 respectively, change in weight z-scores over the first year of life) and (3) numbers of the various combinations of compound fetal alleles formed from the paternally-transmitted *IGF2* rs1077025 allele [8, 9] and the maternally-transmitted *H19* rs2071094 allele [16]. The remaining participants in this analysis (*n* = 567), were used as an internal Validation Cohort. Clinical characteristics of participants in the Discovery and Validation Cohorts are shown in Table 1. The only detectable difference between the two groups was a slightly shorter mean gestational age at delivery in the Validation Cohort of around 2 days.

**Table 1:** Characteristics of the study participants drawn from the Cambridge Baby Growth Study

Characteristic	Discovery Cohort (*n* = 200)	Validation Cohort (*n* = 567)	*p*-value
Age at delivery (years)	33.4 (32.8, 34.0)	33.2 (32.8, 33.6)	0.6
Gestational age at delivery (weeks)	40.1 (39.9, 40.3)	39.8 (39.7, 40.0)	0.02
Parity (n)	1.7 (1.6, 1.8)	1.7 (1.6, 1.8)	0.6
Pre-pregnancy BMI (kg/m^2^)	23.9 (23.2, 24.6)	24.2 (23.8, 24.6)	0.5
Weight gain during pregnancy (kg)	9.2 (8.0, 10.4)	9.0 (8.2, 9.7)	0.8
Week 28 OGTT fasting glucose concentration (mmol/L)	4.3 (4.2, 4.4)	4.3 (4.3, 4.3)	0.9
Week 28 OGTT 60 min. glucose concentration (mmol/L)	6.4 (6.2, 6.7)	6.6 (6.5, 6.8)	0.1
Week 28 fasting insulin concentration (pmol/L)	44.9 (41.7, 48.4)	45.6 (43.7, 47.6)	0.7
Week 28 fasting insulin resistance (HOMA IR)	0.915 (0.850, 0.985)	0.925 (0.886, 0.965)	0.8
Carrying a male fetus (%)	49.2	52.6	0.4
Smoking during pregnancy (%)	2.6	4.9	0.2

Data are mean (95% confidence interval) or counts (%) for those study participants where the information was available. Categorical data was assessed using the χ^2^ test, all other testing was performed using linear regression

### Gene selection and genetics

Genomic DNA was extracted from blood samples or mouth swabs using an Autopure LS Machine (Qiagen Ltd., Crawley, U.K.). The 16 genes that were studied (*DLK1, FAM99A, GNAS, GRB10, H19, IGF2, INS, KCNQ1OT1, MEST, NNAT, PEG3, PEG10, PLAGL1, SGCE, SNRPN, ZIM2*) were chosen because they were all imprinted at some stage of development in certain tissues. They were also all placentally-expressed [17], although by the time in pregnancy that the placenta was fully functional some of them may have been biallelically expressed. The variants that were genotyped were haplotype tag SNPs covering the gene and 20 kb either side of it, identified by Tagger (r^2^ > 0.8 and minor allele frequency > 0.2) from the Centre d’Etude du Polymorphisme Human population (Utah residents with ancestry from Northern and Western Europe) of HapMap Project Build 36 using Haploview [18]. The one exception to this was *IGF2* where 11 of the tagging SNPs were identified by Rodríguez et al. [19]. The SNPs that were genotyped are listed in Table 2. The DNA samples were genotyped using KASPar assays, which are competitive allele-specific PCR SNP genotyping assays using FRET quencher cassette oligonucleotides (designed and performed by LGC Genomics, Hoddesdon, U.K.). The SNP genotypes that were used in this study were consistent with Hardy Weinberg equilibrium (*p* > 0.05 using the χ^2^ test) and had a repeat genotyping discordancy rate of < 1.0%. For each of the 151 SNPs that were successfully designated, genotypes from both parents and their child were used to infer parental allelic transmission to the fetus according to Table 3.

**Table 2:** The SNPs that were genotyped for this study

Gene	No. of SNPs	Single nucleotide polymorphisms
*DLK1*	5	rs12147008, rs7155375, rs10139403, rs1802710, rs7147586
FAM99A*	7	rs4752779, rs4752781, rs11600502, rs10839220, rs11607954, rs1489945, rs7131362
*GNAS*	20	rs6123832, rs965808, rs6128441, rs6026561, rs6026560, rs12625436, rs4810148, rs7271854, rs6100260, rs6026576, rs6128461, rs6123837, rs13831, rs234630, rs919196, rs3730168, rs919197, rs7121, rs234623, rs234621
*GRB10*	17	rs4245555, rs12669770, rs7802879, rs7777754, rs2715129, rs3807549, rs2299150, rs11769934, rs2237441, rs1468450, rs6948959, rs980716, rs2715116, rs737292, rs7794604, rs17133917, rs2237450
*H19*	13	rs2071094, rs2735466, rs7950932, rs217222, rs217727, rs2251375, rs217228, rs3741216, rs1706879, rs2735971, rs7950715, rs7950787, rs12417375
IGF2*	17	rs6578987, *rs734351, *rs4341514, rs680, *rs3741212, *rs11603378, *rs3213216, *rs10770125, *rs3741205, *rs3741206, rs1004446, *rs4320932, *rs3842752, *rs7924316, *rs7483056, *rs6356, *rs10743152
INS*	16	*rs734351, *rs4341514, rs2585, *rs3741212, *rs11603378, *rs3213216, *rs10770125, *rs3741205, *rs3741206, *rs4320932, rs3741211, *rs3842752, *rs7924316, *rs7483056, *rs6356, *rs10743152
KCNQ1OT1*	14	rs231352, rs463924, rs4930005, rs231841, rs10832514, rs756852, rs7128926, rs10832430, rs231361, rs760419, rs9666537, rs7929804, rs10766218, rs6578283
*MEST*	6	rs12706933, rs13234660, rs765205, rs13225903, rs1047456, rs10954272
*NNAT*	3	rs6066671, rs12481150, rs6019103
PEG3*	8	rs17207579, rs11084466, *rs2079877, *rs7256072, *rs382592, rs758752, rs7248098, *rs1860565
*PEG10*	5	rs1568214, rs13073, rs1533831, rs6948099, rs6465422
*PLAGL1*	20	rs12528289, rs9484833, rs1884087, rs10499846, rs2268440, rs6930726, rs7770529, rs2268443, rs2876578, rs9373409, rs2268445, rs17073273, rs9403542, rs9484836, rs6570599, rs2328537, rs9321957, rs6937128, rs9767615, rs2207851
*SGCE*	2	rs1879854, rs7778237
*SNRPN*	12	rs7164801, rs17114774, rs7171320, rs1453556, rs4028398, rs1982981, rs736008, rs9806682, rs7164989, rs8042562, rs12592279, rs8029318
ZIM2*	8	*rs2079877, rs12981689, rs1476830, *rs7256072, *rs382592, *rs1860565, rs2286751, rs10401139

Genes are named according to their HUGO (Human Genome Organisation) abbreviation

*indicates that some of the SNPs are shared with another gene (*IGF2* with *INS, PEG3* with *ZIM2, PLAGL1* with *HYMAI*, *KCNQ1OT1* with *KCNQ1,* and *FAM99A* with *FAM99B and KRTAP5-6*). A total of 155 SNPs were genotyped. The following SNP genotypes failed as either the assay was unsuitable or the genotypes did not pass the quality control procedures: (*GNAS*) rs6123837, (*GRB10*) rs2237450, (*PLAGL1*) rs12528289, (*ZIM2*) rs2286751

**Table 3:** Imputation table of parentally transmitted fetal alleles based on family triad genotypes

Paternal Genotype	Maternal Genotype	Fetal Genotype	Paternally-transmitted Allele	Maternally-transmitted Allele
A/A	A/A	A/A	A	A
A/A	A/a	A/A	A	A
A/A	A/a	A/a	A	a
A/A	a/a	A/a	A	a
A/a	A/A	A/A	A	A
A/a	A/A	A/a	a	A
A/a	A/a	A/A	A	A
A/a	A/a	A/a	Uninformative	Uninformative
A/a	A/a	a/a	a	a
A/a	a/a	A/a	A	a
A/a	a/a	a/a	a	a
a/a	A/A	A/a	a	A
a/a	A/a	A/a	a	A
a/a	A/a	a/a	a	a
a/a	a/a	a/a	a	a

“A” represents the major allele of a SNP and “a” the minor allele based on frequency

### Lipid profiling: Sample extraction and mass spectrometry

The lipid profiles of maternal serum from the Discovery Cohort were analysed using the direct infusion mass spectrometry method that we developed for use with dried blood spots [20, 21], which was originally based on a method for plasma [22]. All solvents were of at least liquid chromatography–mass spectrometry (LC–MS) grade and were purchased from Sigma Aldrich (Gillingham, Dorset, U.K.). All internal standards were obtained from Avanti Polar lipids (through Instruchem, Delfzijn, The Netherlands) except for undecanoic acid and trilaurin (Sigma Aldrich).

Samples were subjected to a balanced randomisation to achieve an approximately equal number of genotypes in each batch (2 × 68 samples and 1 × 64 samples). A serum pool was created by using 5 μL of a random subset of samples, to create the first quality control (QC1). Part of the pooled sample was diluted with phosphate buffered saline (PBS), either mixing the pooled sample 1:1 with PBS (to create QC2) or 1:4 with PBS (to create QC3). From the QC material 15 μL was aliquoted into 1.2 mL Cryovials and stored at − 80 °C prior to use. Quality assurance (QA) samples used were based on commercially available human plasma. Each batch had 6 x blanks, 6 x QAs, 4 x QC1, 4 x QC2, 4 xQC3. The extraction method for the lipids used a Flexus automated liquid handler (Anachem, Milton Keynes, UK) for pipetting. 15 μL serum samples were extracted using *tert*-butyl methyl ether (MTBE), containing the following internal standards: 1,2-di-*O*-octadecyl-glycero-*sn*-3-phosphatidylcholine, 1,2-di-*O*-phytanyl-glycero-*sn*-3-phosphatidylethanolamine, C8-ceramide, N-heptadecanoyl-D-erythro-sphingosylphosporylcholine, undecanoic acid and trilaurin on glass coated 2.4 mL deep well plates (Plate+TM, Esslab, Hadleigh, UK) as described [20]. A 96 head micro-dispenser (Hydra Matrix, Thermo Fisher Ltd., Hemel Hampstead, U.K.) was used to transfer 25 μL of the lipid extract to a glass coated low well plate (Plate+TM, Esslab, Hadleigh, U.K.) and with 90 μL of MS-mix (7.5 mM NH_4_Ac IPA:MeOH (2:1)) using a Hydra Matrix, after which the plate was sealed using Corning aluminium microplate sealing tape (Sigma-Aldrich Company, U.K.) and stored at − 20 °C until analysis.

At 15 °C throughout, 5 μL of sample was dispensed into the mass spectrometer (Thermo Fisher Exactive benchtop orbitrap; Hemel Hempstead, U.K.) using an Advion Triversa Nanomate (Ithaca, New York, U.S.A.) electrospray ioniser under 0.2 psi nitrogen pressure. Ionisation was achieved with a potential of 1.2 kV. Data acquisition started 20 s. after sample aspiration began. After 72 s. of acquisition in positive mode, negative mode was adopted by changing the potential to − 1.5 kV. The spray was maintained for another 66 s.

### Liquid chromatography–mass spectrometry

Lipids were identified using a selected subset of samples analysed using a LTQ Orbitrap Velos Elite coupled to a Surveyor (both Thermo Fisher, Hemel Hampstead, U.K.) LC system as described [21]. The Orbitrap was set-up using the data-dependent precursor selection. Generally the selected masses were isolated with a 1.5 m/z width in the linear ion trap and then fragmented using either linear ion trap with 35% relative collision energy or in the higher-energy collision-induced dissociation collision cell, with a range from 5 to 75% relative collision energy. All spectra were recorded in the Orbitrap set at 100,000 resolution. All species recorded were identified based on accurate mass and subsequently based on accurate mass MS2 spectra and retention time from the LC–MS experiment.

The maternal serum lipid profiles of the Validation Cohort were subsequently analysed by LC–MS rather than by the direct infusion mass spectrometry that was used for the Discovery Cohort [23], using the method described previously [24].

The raw mass spectrometry data were converted to .mzXML format using MS Convert, parsed to R and 50 spectra averaged per mode using XCMS (http://bioconductor.org/packages/release/bioc/html/xcms.html). Additional processing was then conducted using an in-house R script (Peakpicker version 2.0) to select peaks, deisotope and annotate direct-infusion mass spectrometry data sets. Only those lipid species where more than 70% (140) of the samples contained detectable concentrations and showed a linear correlations with diluted QC range were used. Accordingly 173 lipid/triglyceride species were considered for statistical analysis.

### Other assays and calculations

Blood glucose concentrations were measured using a routine glucose oxidase-based method. Maternal plasma insulin concentrations were measured using a DSL ELISA (London, U.K.) run according to the manufacturer’s instructions. Homeostatic model assessment of insulin resistance (HOMA IR) was estimated using the HOMA calculator [25]. Body mass index (BMI) was calculated as the body weight (kg) divided by the height (m) squared.

### Statistical power

In the Discovery Cohort there was 80% statistical power to detect a difference of 1.37 standard deviations in lipid species concentrations for a variant with a minor allele frequency of 0.4 (α = 0.05). The equivalent value for the Validation Cohort was 1.21. There was 80% statistical power in the random-effects meta-analysis with moderate heterogeneity for a small effect size (Cohen’s d) of 0.29 (α = 0.05).

### Statistical analyses

Lipid profiles in the Discovery Cohort were analysed by Partial Least Squares – Discriminant Analysis (PLS-DA), a multivariate dimensionality-reduction tool with awareness of class labels, using Metaboanalyst 3.0 [26]. The peak intensity table from the mass spectrometry was loaded into the software and the default data integrity check accepted. Without data filtering, the log-transformed data was normalised using the median and scaled using mean centring. Fitted models were considered significant if the value of *Q*^2^ (representing the predictive capability of the model, as estimated by cross-validation) was positive [23]. The importance of individual lipid species was quantified via the variable importance in projection (VIP) score, a weighted sum of squares of the PLS-DA loadings that takes into account the amount of explained lipid profile variance for each component, and used to identify individual candidate lipid species (with a threshold VIP score of greater than 2.0 in this study) that may be significantly associated with fetal imprinted gene alleles. These associations (and those with maternal genotypes) were then tested using linear regression models in both the Discovery and Validation Cohorts. Analysis of the associations in the full cohort was by meta-analysis rather than combining results from both sub-Cohorts into one because of the different laboratory methods used to assess the lipid profiles. The meta-analysis was analysed using the Der Simonian-Laird approach [27] for random effects models implemented into the R Metacor package, version 1.0–2 (https://cran.r-project.org/packages/metacor/index.html). Additional analyses were then performed to assess potential imprinting effects on the associations, by testing associations between the lipid species and the maternal genotypes, the maternal untransmitted allele and the fetal imprinted allele transmitted from the parent whose allele did not produce significant fitted models in Metaboanalyst.

Categorical and ordinal data were analysed using the χ^2^ or Fisher’s exact tests, or logistic regression. Associations with *p* < 0.05 were considered significant throughout. Apart from the use of Metaboanalyst, and the meta-analyses which were performed in R (version 3.2.2; https://cran.r-project.org/bin/windows/base/), all other statistical analyses were performed in Stata (version 13.1; StataCorp LP, College Station, TX, U.S.A.).

## Results

### Associations between fetal imprinted gene alleles and the maternal lipid profile around week 15 of pregnancy

The associations between the peak intensities of individual lipid species (that produced significant fitted models in the PLS-DA analysis of the Discovery Cohort) and fetal imprinted gene alleles are shown in Table 4. The most significant fetal imprinted gene allele associations both related to TG(44:1), namely paternally-transmitted *H19* rs7950932 and maternally-transmitted *FAM99A* rs7131362. Of the associations in this list, not all of which reached statistical significance, there was a predominance of associations of species with maternally-expressed fetal alleles in the Discovery Cohort analyses (*p* = 0.01). Due to this we also tested associations between the same species and both the maternal genotype (Table 5) and the untransmitted maternal allele (Table 6) to investigate whether the associations were most likely fetally- or maternally-mediated. All other associations were considered non-significant (data not shown). To assess whether the associations highlighted in Table 4 were parent-of-origin effects we also tested associations between the same species and fetal alleles transmitted from the parent whose allele did not produce significant fitted models in the PLS-DA analysis of the Discovery Cohort (Table 7). Fetal *H19* rs7950932 alleles transmitted from both parents showed significant associations with maternal TG(44:1) peak intensity (Tables 4 and 7), and we also found significant associations with the fetal rs7950932 genotype (Discovery Cohort *R* = 0.17, *p* = 0.01, *n* = 191; Validation cohort *R* = 0.10, *p* = 0.08, *n* = 220; meta-analysis *R* = 0.13 (0.04–0.23), *p* = 3.6 × 10^− 3^).

**Table 4:** Associations between fetal genotypes and candidate lipid species’ peak intensity

SNP	Gene	Parental Transmission	Lipid	VIP Score of lipid species*	Discovery Cohort			Validation Cohort			Meta-Analysis	
					R	*p*-value	n	R	*p*-value	n	R	*p*-value
rs10839220	*KRTAP5–6*	Maternal	TG(44:1)	3.4	0.175	0.03	163	0	0.96	197	0.09 (− 0.09, 0.25)	0.17
rs10839220	*KRTAP5–6*	Maternal	LysoPCP(18:0)	2.8	0.243	1.8 × 10^− 3^	163	0.092	0.16	233	0.16 (0.01, 0.31)	0.02
rs10839220	*KRTAP5–6*	Maternal	PE(34:0)	2.2	0.279	3.2 × 10^−4^	163	0.014	0.85	233	0.15 (−0.12, 0.39)	0.14
rs10839220	*KRTAP5–6*	Maternal	DG(36:3)	2.1	0.271	4.6 × 10^−4^	163	0.118	0.09	209	0.19 (0.04, 0.34)	7.4 × 10^−3^
rs4752779	*FAM99B*	Maternal	TG(44:1)	3.2	0.161	0.03	173	0.030	0.67	201	0.09 (−0.04, 0.22)	0.08
rs7131362	*FAM99A*	Maternal	TG(44:1)	4.7	0.257	9.7 × 10^−4^	161	0.114	0.12	190	0.18 (0.04, 0.32)	6.2 × 10^−3^
rs7131362	*FAM99A*	Maternal	TG(48:0)	2.1	0.197	0.01	161	0	0.95	217	0.10 (−0.10, 0.28)	0.17
rs17073273	*PLAGL1*	Maternal	TG(44:1)	3.5	0.181	0.02	175	0.014	0.83	206	0.09 (−0.07, 0.25)	0.13
rs17073273	*PLAGL1*	Maternal	TG(55:9)	2.7	0.234	1.8 × 10^−3^	175	0.079	0.23	235	0.15 (0.00, 0.30)	0.03
rs17073273	*PLAGL1*	Maternal	TG(48:0)	2.5	0.226	2.6 × 10^−3^	175	0.035	0.59	235	0.13 (−0.06, 0.31)	0.09
rs2268445	*PLAGL1*	Maternal	TG(44:1)	3.6	0.189	0.01	176	0.035	0.62	212	0.11 (−0.04, 0.26)	0.08
rs7950932	*H19*	Paternal	TG(44:1)	2.6	0.167	0.02	183	0.117	0.10	203	0.14 (0.04, 0.24)	2.3 × 10^−3^

Only those lipid species that tagged components with significant fitted models in Metaboanalyst are shown

*in the PLSDA component with a significant Q^2^ in the Discovery Cohort dataset

**Table 5:** Associations between maternal imprinted gene SNP genotypes and candidate lipid species’ peak intensity

SNP	Gene	Lipid	Discovery Cohort			Validation Cohort			Meta-Analysis	
			R	*p*-value	n	R	*p*-value	n	R	*p*-value
rs10839220	*KRTAP5–6*	TG(44:1)	0.157	0.04	179	0.017	0.78	225	0.08 (−0.05, 0.22)	0.12
rs10839220	*KRTAP5–6*	LysoPCP(18:0)	0.205	5.8 × 10^−3^	179	0	0.92	267	0.10 (−0.10, 0.29)	0.17
rs10839220	*KRTAP5–6*	PE(34:0)	0.194	9.2 × 10^−3^	179	0.051	0.41	267	0.12 (− 0.02, 0.25)	0.05
rs10839220	*KRTAP5–6*	DG(36:3)	0.224	2.6 × 10^−3^	179	0.062	0.34	240	0.14 (−0.02, 0.29)	0.04
rs4752779	*FAM99B*	TG(44:1)	0.102	0.17	180	0.045	0.51	227	0.07 (−0.03, 0.17)	0.08
rs7131362	*FAM99A*	TG(44:1)	0.222	2.9 × 10^− 3^	178	0.041	0.54	221	0.13 (−0.05, 0.30)	0.08
rs7131362	*FAM99A*	TG(48:0)	0.150	0.05	178	0.030	0.64	251	0.08 (−0.03, 0.20)	0.08
rs17073273	*PLAGL1*	TG(44:1)	0.042	0.56	188	0.033	0.61	234	0.04 (−0.06, 0.13)	0.23
rs17073273	*PLAGL1*	TG(55:9)	0.125	0.09	188	0.117	0.06	267	0.12 (0.03, 0.21)	5.2 × 10^−3^
rs17073273	*PLAGL1*	TG(48:0)	0.084	0.25	188	0.057	0.35	267	0.07 (− 0.02, 0.16)	0.07
rs2268445	*PLAGL1*	TG(44:1)	0.046	0.54	187	0.071	0.28	231	0.06 (−0.04, 0.16)	0.11

Only those lipid species that tagged components with significant fitted models in Metaboanalyst using maternally-transmitted fetal variants are shown

**Table 6:** Associations between maternal untransmitted alleles and candidate lipid species’ peak intensity

SNP	Gene	Lipid	Discovery Cohort			Validation Cohort			Meta-Analysis	
			R	*p*-value	n	R	*p*-value	n	R	*p*-value
rs10839220	*KRTAP5–6*	TG(44:1)	0.113	0.16	154	0.067	0.38	179	0.09 (−0.02, 0.19)	0.05
rs10839220	*KRTAP5–6*	LysoPCP(18:0)	0.095	0.24	154	0.114	0.10	213	0.11 (0, 0.21)	0.02
rs10839220	*KRTAP5–6*	PE(34:0)	0.068	0.54	154	0.042	0.40	213	0.05 (−0.05, 0.15)	0.16
rs10839220	*KRTAP5–6*	DG(36:3)	0.122	0.13	154	0	0.98	190	0.06 (−0.06, 0.17)	0.18
rs4752779	*FAM99B*	TG(44:1)	0.020	0.81	161	0.054	0.47	185	0.04 (−0.07, 0.14)	0.24
rs7131362	*FAM99A*	TG(44:1)	0.119	0.14	153	0.050	0.51	175	0.08 (−0.03, 0.19)	0.07
rs7131362	*FAM99A*	TG(48:0)	0.080	0.43	153	0.065	0.26	198	0.07 (−0.03, 0.18)	0.09
rs17073273	*PLAGL1*	TG(44:1)	0.131	0.09	170	0.024	0.74	201	0.07 (−0.03, 0.18)	0.09
rs17073273	*PLAGL1*	TG(55:9)	0	0.98	170	0.123	0.06	228	0.07 (−0.05, 0.19)	0.13
rs17073273	*PLAGL1*	TG(48:0)	0.094	0.22	170	0.067	0.31	228	0.08 (−0.02, 0.18)	0.06
rs2268445	*PLAGL1*	TG(44:1)	0.092	0.23	172	0.022	0.76	201	0.05 (−0.05, 0.16)	0.15

Only those lipid species that tagged components with significant fitted models in Metaboanalyst using maternally-transmitted fetal variants are shown

**Table 7:** Associations between candidate lipid species’ peak intensity and alleles transmitted from the other parent than those with significant fitted models

SNP	Gene	Parental Transmission	Lipid	Discovery Cohort			Validation Cohort			Meta-Analysis	
				R	*p*-value	n	R	*p*-value	n	R	*p*-value
rs10839220	*KRTAP5–6*	Paternal	TG(44:1)	0.12	0.14	161	0.08	0.26	200	0.10 (−0.01, 0.20)	0.03
rs10839220	*KRTAP5–6*	Paternal	LysoPCP(18:0)	0.07	0.40	161	0.12	0.07	235	0.07 (−0.03, 0.17)	0.08
rs10839220	*KRTAP5–6*	Paternal	PE(34:0)	0.10	0.20	161	0.11	0.10	235	0.11 (0.01, 0.20)	0.02
rs10839220	*KRTAP5–6*	Paternal	DG(36:3)	0.05	0.53	161	0.18	8.4 × 10^−3^	213	0.12 (−0.01, 0.25)	0.03
rs4752779	*FAM99B*	Paternal	TG(44:1)	0.03	0.66	174	0.04	0.61	205	0.04 (−0.07, 0.14)	0.25
rs7131362	*FAM99A*	Paternal	TG(44:1)	0.05	0.57	159	0.01	0.86	204	0.03 (−0.08, 0.13)	0.30
rs7131362	*FAM99A*	Paternal	TG(48:0)	0.03	0.68	159	0.03	0.64	232	0.03 (−0.07, 0.13)	0.26
rs17073273	*PLAGL1*	Paternal	TG(44:1)	0.14	0.07	174	0.08	0.26	216	0.11 (0.01, 0.20)	0.02
rs17073273	*PLAGL1*	Paternal	TG(55:9)	0.13	0.08	174	0.03	0.66	248	0.07 (−0.03, 0.17)	0.08
rs17073273	*PLAGL1*	Paternal	TG(48:0)	0.19	0.01	174	0.10	0.10	248	0.14 (0.04, 0.23)	2.4 × 10^−3^
rs2268445	*PLAGL1*	Paternal	TG(44:1)	0.14	0.06	177	0.14	0.04	218	0.14 (0.04, 0.24)	2.7 × 10^−3^
rs7950932	*H19*	Maternal	TG(44:1)	0.09	0.21	183	0.09	0.22	209	0.09 (−0.01, 0.19)	0.04

### Associations between the abundance of TG(44:1) at around week 15 of pregnancy and clinically significant parameters

The species most heavily represented in the list of associations with fetal imprinted gene variant alleles by the VIP scores was TG(44:1), a monounsaturated triglyceride. It is also the triglyceride involved in the two most significant associations between fetal imprinted gene alleles and triglycerides in analyses of lipid species where PLS-DA analysis of the Discovery Cohort data led to significant fitted models (Table 4). To investigate whether this isoform was clinically relevant in our population we tested associations between its peak intensities, maternal pre-pregnancy BMI and indices derived from the OGTT (Table 8). Of these in the two sub-cohorts the strongest associations were with fasting glucose concentrations and HOMA IR, both of which were positively related to the abundance of TG(44:1). In turn week 28 fasting glucose concentrations (odds ratio (OR) 4.8 × 10^10^ (8.5 × 10^8^, 2.7 × 10^12^), *p* = 5.7 × 10^− 33^, *n* = 1083) and HOMA IR (OR 7.7 (4.8, 12.1), *p* = 2.7 × 10^− 18^, *n* = 1059) were also both positively associated with GDM in the Cambridge Baby Growth Study as a whole. In addition they were both positively associated with baby’s birth weight (fasting glucose concentrations: standardised β = 0.116, *p* = 3.7 × 10^− 6^, *n* = 813; HOMA IR: standardised β = 0.065, *p* = 0.048, n = 813; both adjusted for gestational age at birth, sex, parity and maternal BMI).

**Table 8:** Associations between week 15 TG (44:1) peak intensities and biomarkers derived from the week 28 OGTT

Biomarker	Discovery Cohort			Validation Cohort			Meta-Analysis	
	R	*p*-value	n	R	*p*-value	n	R	*p*-value
Pre-pregnancy BMI	−0.10	0.21	170	−0.06	0.31	356	−0.07 (− 0.16, 0.01)	0.05
Fasting glucose concentration	0.04	0.54	195	0.11	0.02	478	0.09 (0.01, 0.16)	9.9 × 10^−3^
Glucose concentration 60 min. post 75 g load	0.16	0.03	195	0.05	0.26	478	0.09 (−0.01, 0.19)	0.04
Fasting insulin concentration	0.03	0.65	189	0.10	0.03	476	0.08 (0, 0.16)	0.02
HOMA IR	0.03	0.65	189	0.12	8.8 × 10^−3^	475	0.09 (0.01, 0.17)	0.01
HOMA B	0.03	0.72	189	0.05	0.26	475	0.04 (−0.03, 0.12)	0.13

## Discussion

In this detailed investigation of the associations between fetal imprinted gene alleles and the circulating maternal lipid profile around week 15 of pregnancy, we found several significant associations with particular triglyceride isoform peak intensities in the maternal circulation. There was an overrepresentation of maternally-transmitted fetal alleles in the list of associations with fitted models in the PLS-DA analysis. Whilst unexpected this may relate to an influence of triglycerides on fetal growth [13] and the extra influence that maternal genes may have on the resulting offspring birth weight in particular in comparison to paternal genes [28]. In previous studies we observed an influence of fetal imprinted genes on maternal glucose concentrations in pregnancy, although these were much later in pregnancy around week 28 [8, 9]. Results from the current study provide the first evidence for a potential role for fetal imprinted genes in modifying maternal metabolism, in particular circulating levels of certain lipid species, relatively early in pregnancy.

The strongest association was detected with the abundance of maternal circulating TG(44:1), which may include configurations 15:0/15:0/14:1 and/or 15:1/14:0/15:0 [29]. This lipid species was linked to the development of insulin resistance and type 2 diabetes in the Framingham Offspring Study [30]. Importantly, in the current study this isoform was associated with paternally-transmitted fetal rs7950932 (Table 4). This variant is one of the single nucleotide polymorphisms (SNPs) that was used to tag *H19*, which is generally expressed only from the maternally-transmitted allele [31]. However there was also an association between the maternally-transmitted rs7950932 allele and TG(44:1) (Table 7), possibly resulting from biallelic expression in some tissues at this stage of development (which would be consistent with there also being a significant association with the fetal rs7950932 genotype). In other tissues, however, the alleles may have been expressed only from the paternally-transmitted allele: this interpretation being consistent with the association with the paternally-transmitted allele having a higher regression coefficient (and being more statistically significant) than that with the maternally-transmitted allele. Although when expressed in an imprinted fashion *H19* is expressed only from the maternal allele, the rs7950932 locus is located in the proximity of *H19*’s differentially methylated region [32] which helps regulate paternally-expressed *IGF2* as well as *H19* expression [33], making the association with the paternally-transmitted allele plausible. Interestingly the abundance of TG(44:1) in maternal serum around week 15 of pregnancy correlated with subsequent (week 28) fasting glucose concentrations and HOMA IR in our population, which in turn were associated with both GDM and offspring birth weight. We have previously found several paternally-transmitted fetal *IGF2* variants that were associated with late pregnancy glucose concentrations [8, 9]. Together all these results suggest that the associations between fetal rs7950932 alleles and week 15 maternal TG(44:1) levels may be related to both *IGF2* and *H19* expression*.*

The second strongest association in our population was between fetal rs7131362, a downstream variant of the *FAM99A* gene, and the abundance of maternal week 15 TG(44:1) again. The association was with the maternally-transmitted fetal allele, and with the associations having larger regression coefficients (and greater statistical significance) than those of the equivalent associations with the maternal genotype and the untransmitted maternal alleles, it appears to be directly related to the transmitted fetal allele itself rather than the maternal gene per se. The lack of association with the paternally-transmitted allele is consistent with the fetal effect being modified by imprinting. This variant is in a chromosomal region populated by reciprocally imprinted genes a number of which are maternally-expressed [34]. Although a parent-of-origin effect of this variant on maternal circulating lipid levels has not been evident before, other published studies suggest why such an association may be plausible. Data from the 1000 Genomes project [35] shows a degree of linkage disequilibrium between rs7131362 and rs2334499 (D’ = 0.86), whose maternally-transmitted allele has been associated with risk of type 2 diabetes [36] (with the protective allele in [36] being in linkage disequilibrium with the allele associated with the lower TG(44:1) peak intensity in the present study). In recent years it has become apparent that there is a strong correlation between genetic risk variants for type 2 diabetes and those for GDM [37]. As described above, in our population the abundance of the triglyceride isoform TG(44:1) was associated with factors themselves associated with risk of GDM.

Even the most significant associations found in this study should be considered preliminary in nature. The main limitation of the study is that it was only powered to detect associations with large effect sizes. Nevertheless the main associations in our population appear biologically plausible based on current knowledge in the published literature. The associations need external validation in other cohorts, rather than the internal validation that was used in this study, but they will be useful for meta-analyses once studies in other populations have been completed. Our results suggest that associations with TG(44:1) in pregnancy may be particularly important.

## Conclusion

The results of this study suggest that the interaction between the fetal imprinted genotype and maternal metabolism may be detected early in pregnancy through alterations in serum levels of clinically relevant triglycerides in the maternal circulation. This result is remarkable because it suggests that triglycerides, traditionally regarded as merely energy storage molecules, may be involved in the metabolic signalling that controls fetal development. However further validation in more highly powered studies is required to establish this.

## Abbreviations

BMI: Body mass index
GDM: Gestational diabetes mellitus
HOMA B: Homeostasis model assessment of β-cell function
HOMA IR: Homeostasis model assessment of insulin resistance
LC^_^MS: Liquid chromatography–mass spectrometry
MTBE: Methyl *tert*-butyl ether
OGTT: Oral glucose tolerance test
OR: Odds ratio
PBS: Phosphate-buffered saline
PLS-DA: Partial least squares discriminant analysis
QA: Quality assurance
QC: Quality control
SNP: Single nucleotide polymorphism
TG(44:1): Triglyceride with 44 carbon atoms and 1 double bond
VIP: Variable importance in projection *Individual lipid species are abbreviated according to LIPID MAPS (* *http://www.lipidmaps.org/data/classification/lipid_cns.html* *)*

## References

[1] Haig D, Westoby M (1989). Parent-specific gene expression and the triploid endosperm. Am Nat.

[2] Haig D (1993). Genetic conflicts in human pregnancy. Q Rev Biol.

[3] Reik W, Constância M, Fowden A, Anderson N, Dean W, Ferguson-Smith A (2003). Regulation of supply and demand for maternal nutrients in mammals by imprinted genes. J Physiol.

[4] Haig D (1996). Placental hormones, genomic imprinting, and maternal–foetal communication. J Evol Biol.

[5] Petry CJ, Ong KK, Dunger DB (2007). Does the foetal genotype affect maternal physiology during pregnancy?. Trends Mol Med.

[6] Petry CJ, Beardsall K, Dunger DB (2014). The potential impact of the foetal genotype on maternal blood pressure during pregnancy. J Hypertens.

[7] Petry CJ, Evans ML, Wingate DL, Ong KK, Reik W, Constância M (2010). Raised late pregnancy glucose concentrations in mice carrying pups with targeted disruption of H19delta13. Diabetes.

[8] Petry CJ, Seear RV, Wingate DL, Manico L, Acerini CL, Ong KK (2011). Associations between paternally transmitted fetal IGF2 variants and maternal circulating glucose concentrations in pregnancy. Diabetes.

[9] Petry CJ, Mooslehner K, Prentice P, Hayes MG, Nodzenski M, Scholtens DM, Hughes IA, Acerini CL, Ong KK, Lowe WL, Dunger DB (2017). Associations between a fetal imprinted gene allele score and late pregnancy maternal glucose concentrations. Diabetes Metab.

[10] Petry CJ, Sanz Marcos N, Pimentel G, Hayes MG, Nodzenski M, Scholtens DM (2016). Associations between fetal imprinted genes and maternal blood pressure in pregnancy. Hypertension.

[11] Simopoulos AP (2010). Genetic variants in the metabolism of omega-6 and omega-3 fatty acids: their role in the determination of nutritional requirements and chronic disease risk. Exp Biol Med (Maywood).

[12] Lattka E, Koletzko B, Zeilinger S, Hibbeln JR, Klopp N, Ring SM (2013). Umbilical cord PUFA are determined by maternal and child fatty acid desaturase (FADS) genetic variants in the Avon longitudinal study of parents and children (ALSPAC). Br J Nutr.

[13] Ghio A, Bertolotto A, Resi V, Volpe L, Di Cianni G (2011). Triglyceride metabolism in pregnancy. Adv Clin Chem.

[14] Schaefer-Graf UM, Graf K, Kulbacka I, Kjos SL, Dudenhausen J, Vetter K (2008). Maternal lipids as strong determinants of foetal environment and growth in pregnancies with gestational diabetes mellitus. Diabetes Care.

[15] Vrijkotte TG, Krukziener N, Hutten BA, Vollebregt KC, van Eijsden M, Twickler MB (2012). Maternal lipid profile during early pregnancy and pregnancy complications and outcomes: the ABCD study. J Clin Endocrinol Metab.

[16] Petry CJ, Seear RV, Wingate DL, Acerini CL, Ong KK, Hughes IA (2011). Maternally transmitted foetal H19 variants and associations with birth weight. Hum Genet.

[17] Frost JM, Moore GE (2010). The importance of imprinting in the human placenta. PLoS Genet.

[18] Barrett JC, Fry B, Maller J, Daly MJ (2005). Haploview: analysis and visualization of LD and haplotype maps. Bioinformatics.

[19] Rodríguez S, Gaunt TR, O’Dell SD, Chen XH, Gu D, Hawe E (2004). Haplotypic analyses of the IGF2-INS-TH gene cluster in relation to cardiovascular risk traits. Hum Mol Genet.

[20] Koulman A, Prentice P, Wong MC, Matthews L, Bond NJ, Eiden M (2014). The development and validation of a fast and robust dried blood spot based lipid profiling method to study infant metabolism. Metabolomics.

[21] Prentice P, Koulman A, Matthews L, Acerini CL, Ong KK, Dunger DB (2015). Lipidomic analyses, breast- and formula-feeding, and growth in infants. J Pediatr.

[22] Graessler J, Schwudke D, Schwarz PE, Herzog R, Shevchenko A, Bornstein SR (2009). Top-down lipidomics reveals ether lipid deficiency in blood plasma of hypertensive patients. PLoS One.

[23] Lu L, Koulman A, Petry CJ, Jenkins B, Matthews L, Hughes IA (2016). An unbiased lipidomics approach identifies early second trimester lipids predictive of maternal glycemic traits and gestational diabetes mellitus. Diabetes Care.

[24] Koulman A, Woffendin G, Narayana VK, Welchman H, Crone C, Volmer DA (2009). High-resolution extracted ion chromatography, a new tool for metabolomics and lipidomics using a second-generation orbitrap mass spectrometer. Rapid Commun Mass Spectrom.

[25] Levy JC, Matthews DR, Hermans MP (1998). Correct homeostasis model assessment (HOMA) evaluation uses the computer program. Diabetes Care.

[26] Xia J, Sinelnikov IV, Han B, Wishart DS (2015). MetaboAnalyst 3.0 - making metabolomics more meaningful. Nucleic Acids Res.

[27] Schulze R (2004). Meta-analysis: a comparison of approaches.

[28] Rice F, Thapar A (2010). Estimating the relative contributions of maternal genetic, paternal genetic and intrauterine factors to offspring birth weight and head circumference. Early Hum Dev.

[29] Roberts LD, Murray AJ, Menassa D, Ashmore T, Nicholls AW, Griffin JL (2011). The contrasting roles of PPARδ and PPARγ in regulating the metabolic switch between oxidation and storage of fats in white adipose tissue. Genome Biol.

[30] Rhee EP, Cheng S, Larson MG, Walford GA, Lewis GD, McCabe E (2011). Lipid profiling identifies a triacylglycerol signature of insulin resistance and improves diabetes prediction in humans. J Clin Invest.

[31] Lustig O, Ariel I, Ilan J, Lev-Lehman E, De-Groot N, Hochberg A (1994). Expression of the imprinted gene H19 in the human fetus. Mol Reprod Dev.

[32] Sandovici I, Kassovska-Bratinova S, Vaughan JE, Stewart R, Leppert M, Sapienza C (2006). Human imprinted chromosomal regions are historical hot-spots of recombination. PLoS Genet.

[33] Vu TH, Li T, Nguyen D, Nguyen BT, Yao XM, Hu JF, Hoffman AR (2000). Symmetric and asymmetric DNA methylation in the human IGF2-H19 imprinted region. Genomics.

[34] Smith AC, Choufani S, Ferreira JC, Weksberg R (2007). Growth regulation, imprinted genes, and chromosome 11p15.5. Pediatr Res.

[35] Auton A, Brooks LD, Durbin RM, Garrison EP, Kang HM, 1000 Genomes Project Consortium (2015). A global reference for human genetic variation. Nature.

[36] Kong A, Steinthorsdottir V, Masson G, Thorleifsson G, Sulem P, Besenbacher S (2009). Parental origin of sequence variants associated with complex diseases. Nature.

[37] Petry CJ, Petry CJ (2014). Genetic risk factors for gestational diabetes. Gestational diabetes: origins, complications and treatment.

